# Porous Silicon Based Resonant Mirrors for Biochemical Sensing

**DOI:** 10.3390/s8106549

**Published:** 2008-10-23

**Authors:** Edoardo De Tommasi, Luca De Stefano, Ilaria Rea, Valentina Di Sarno, Lucia Rotiroti, Paolo Arcari, Annalisa Lamberti, Carmen Sanges, Ivo Rendina

**Affiliations:** 1 Institute for Microelectronics and Microsystems – Unit of Naples – National Council of Research, Via P. Castellino 111, 80131 Napoli, Italy; 2 Department of Physical Sciences, University of Naples “Federico II”, Via Cinthia, 80126 Naples, Italy; 3 Department of Organic Chemistry and Biochemistry, University of Naples “Federico II”, Via Cinthia, 80128 Napoli, Italy; 4 Department of Biochemistry and Medical Biotechnologies, University of Naples “Federico II”, Via S. Pansini 5, 80131 Napoli, Italy

**Keywords:** DNA Optical Biosensors, Porous Silicon, Resonant Mirrors

## Abstract

We report on our preliminary results in the realization and characterization of a porous silicon (PSi) resonant mirror (RM) for optical biosensing. We have numerically and experimentally studied the coupling between the electromagnetic field, totally reflected at the base of a high refractive index prism, and the optical modes of a PSi waveguide. This configuration is very sensitive to changes in the refractive index and/or in thickness of the sensor surface. Due to the high specific area of the PSi waveguide, very low DNA concentrations can be detected confirming that the RM could be a very sensitive and label-free optical biosensor.

## 1. Introduction

Optical label-free biosensors are ideal candidates for high throughput screening in the prevention of bio-threat agents or social illnesses: all the different configurations proposed in the literature, such as optical microcantilever [1], interferometric devices [2, 3], and also the recent photonic crystals geometries [4], show very high sensitivities in the recognition of specific molecules. The detection of analytes present in complex mixtures in ultra-low concentration, such as viruses in human blood, is possible due to two key factors: the utilization of bioprobes, properly linked on the sensor surface, and an optimized design to maximize the interaction between the biological matter and the electromagnetic field.

Resonant Mirrors (RMs) are optical sensors which exploit the evanescent fields features to probe changes in refractive index and thickness taking place on their surface after exposure to gaseous or liquid substances. From the optical point of view, RMs are refractometric sensing devices similar to grating coupler sensors: they are characterized by the high sensitivity typical of waveguiding devices, and, on the other hand, by the simple scheme of Surface Plasmon Resonance (SPR) sensors [5]. In RMs, in fact, incident light gives rise to an evanescent wave at the interface between a prism and an optical waveguide; the coupling of light into the waveguide occurs only at those specific incident angles for which the propagation constant of the evanescent wave matches that of a waveguide mode. This matching produces a dip in the angular spectrum of the reflected light, so that each change in the refractive index and-or in thickness at the sensor surface produces a corresponding shift in the position of the dip [5, 6].

In this work, we report on the realization of a porous silicon (PSi) based RM prototype on chip, whose modal properties and characteristic angular resonances have been both numerically computed and experimentally measured.

PSi is by far one of the most intriguing material in optical sensing: the refractive index is widely tuneable, namely between the silicon refractive index and that of the air, and its specific surface is very large, up to 500 m^2^ cm^-3^. Due to these characteristics, lot of optical structures, such as rugate filters [7] and microcavities [8] have been proposed in literature for biosensing. Recently, a PSi waveguide biosensor have been theoretically and experimentally demonstrated [9]. We have studied and reported in this paper the feasibility of such biosensor for DNA-DNA hybridization experiment.

## 2. Experimental

A highly doped p+-silicon, <100> oriented, 0.01 Ω·cm resistivity, 400 μm thick was used as substrate in the waveguide fabrication. The structure was obtained by electrochemical etching of crystalline silicon in a HF-based solution (50 wt. % HF:ethanol = 3:7) at room temperature. A current density of 10 mA/cm^2^ was applied for 18.9 s to produce the core layer of thickness 2.5 μm and porosity 65 %, while a current density of 109 mA/cm^2^ was applied for 13.6 s in the case of the cladding layer with thickness 2.5 μm and porosity 78 %. These porosities correspond to a core and cladding refractive indexes of 1.749 and 1.384, respectively, calculated by the Bruggeman model [10] at a wavelength of 785 nm. The device was then fully oxidized in pure O_2_ by a two step thermal treatment (400 °C for 30 min and 900 °C for 15 min).

**Figure 1.**
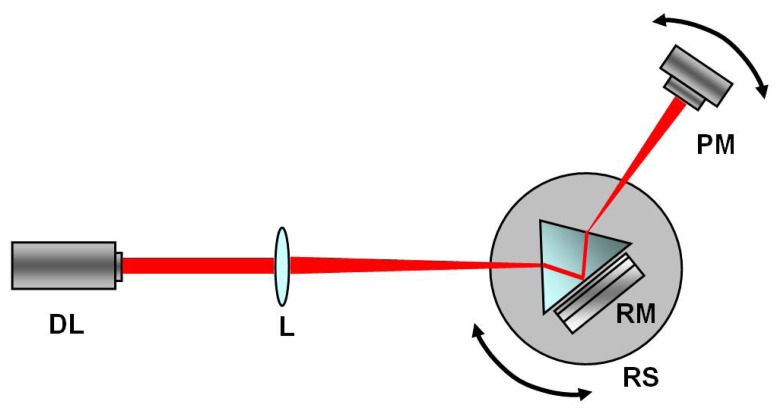
Experimental setup. DL: diode laser emitting s-polarized radiation at 785 nm; L: lens with focal length f=7.5 cm; RM: resonant mirror; RS: rotation stage; PM: power meter on an independent rotation stage.

In Figure 1 a wide view of our experimental set-up is shown. RM is mounted on a rotation stage (Melles Griot, model 07 TRS 501); the light source is a diode laser emitting s-polarized radiation at λ=785 nm (CrystaLaser, model RCL-080-785-5); the laser beam exiting the prism is collected by a photometer (Newport, model 1830-C). The waveguide has been brought in close contact with one side of an SF6 prism (n=1.784), still allowing the presence of a sub-micrometric air layer acting as a coupling zone between the prism and the waveguide.

**Figure 2.**
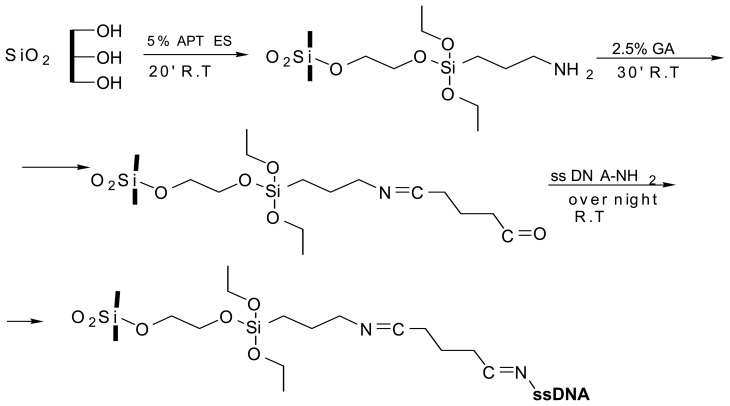
Schematic of the functionalization process, from the oxidized PSi chip to the covalent attachment of the DNA single strands.

The covalent bond of the DNA single strand (5'-GGACTTGCCCGAATCTACGTGTCC-3') on the PSi surface is based on a three steps functionalization process constituted by a chemical passivation of the surfaces, as it is shown in Figure 2 (all the chemicals used in the present study were from Sigma). We have treated the surfaces with a proper chemical linker, the aminopropyltriethoxysilane (APTES). Samples have been rinsed by dipping in a 5 % solution of APTES and a hydroalcoholic mixture of water and methanol (1:1) for 20 min at room temperature. After the reaction time, we have washed the chips by deionized water and methanol, and dried in N_2_ stream. The silanized devices were then baked at 100 °C for 10 min. To create a surface able to link the amino group of the biological probes, we have thus immersed the chips in a 2.5% glutaraldehyde (GA) solution in 20 mM HEPES buffer (pH 7.4) for 30 min, and then rinsed it in deionized water and finally dried in N_2_ stream. The GA reacts with the amino groups on the silanized surface and coats the internal surface of the pores with another thin layer of molecules. All these reaction steps have been monitored by means of FT-IR spectroscopy to verify the presence of the characteristic peaks of the organic linkers. The main characteristic peaks of Si-O-Si (1040 cm^-1^) and Si-OH bonds (3740 and 1646 cm^-1^) are present, after oxidation, on the PSi. After the silanization process, the APTES characteristic peaks of the ethylic (at 1626, 1529 and 1379 cm^-1^), amino (at 1060 cm^-1^) and also the – CH (at 2908 and 2851 cm^-1^) groups are well evident. Finally, after the GA treatment, the characteristic imine group (at 1627 cm-1) due to the reaction with APTES is easily recognized.

The PSi surface was covered overnight at room temperature with a 50 μM DNA probe solution (30 μL). For each measurement, complementary (5'-GGACACGTAGATTCGGGCAAGTCC-3') and non-complementary (5'-CACTGTACGTGCGAATTAGGTGAA-3') DNA (10 μL) were spotted on the chip and incubated for two hours. Before optical measurements, all samples have been extensively rinsed in deionized water to remove the excess of biological matter.

## 3. Results and Discussion

**Figure 3.**
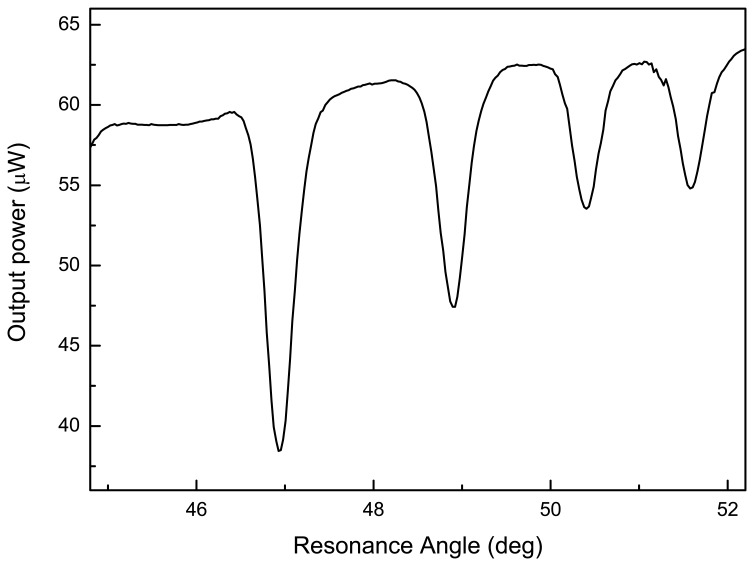
Resonant peaks of the waveguide, corresponding to four guided modes in TE polarization at 785 nm, after functionalization with APTES and GA.

The PSi waveguide, once oxidized in order to reduce the scattering losses [11] and to allow the bond with biological linkers, has been characterized by means of “m lines” technique, which determines the modal behavior of the waveguide at different wavelengths and estimates the refractive indices *n_c_* and *n_b_* for the core an the buffer layers [12]. In Figure 3, is reported a standard m-lines spectrum due to the excitation of four adjacent modes in transverse electric (TE) polarization of the electromagnetic field at 785 nm: by measuring the coupling angles position is possible to quantify the core and cladding thicknesses and refractive indexes. We have obtained the refractive indexes of core and buffer and the thickness of the core equal to *n_c_* = 1.370 ± 0.001, *n_b_* = 1.18 ± 0.01, and d_c_ = 2.71 ± 0.01 μm respectively. It is important to notice the strong reduction of the refractive indexes after the thermal oxidation process, mainly due to the substitution of silicon with silicon dioxide.

**Figure 4.**
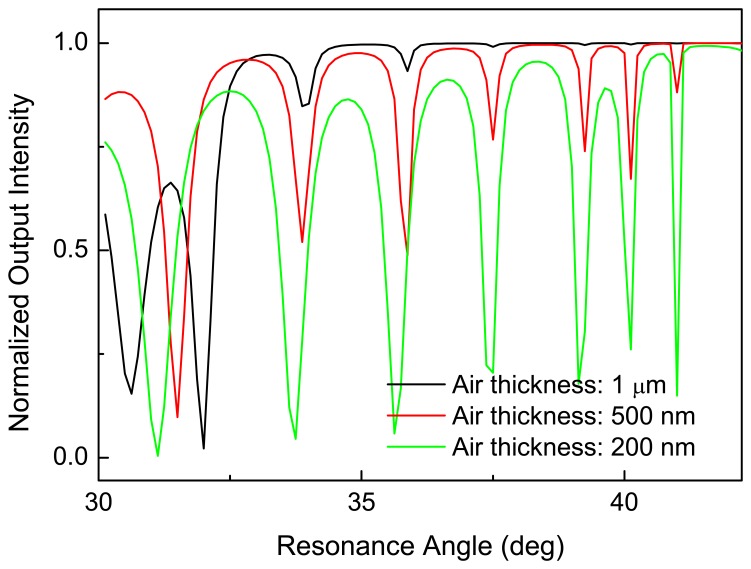
Numerical simulations of RM spectra as a function of the air gap thickness

The close contact between the prism and the waveguide still allows the presence of a sub-micrometric air layer where the evanescent fields coming from the prism and the waveguide can overlap, thus transferring the light energy from the prism to the waveguide. The thickness of this air layer strongly affects the efficiency of the coupling of the incoming light into the waveguide. In Figure 4, some simulations of RM spectra corresponding to different air thicknesses are shown: the lower the thickness, the stronger the coupling of the evanescent wave with the waveguide. The dips present in the total reflection zone (i.e. for incident angle θ*_i_* > 34° ), correspond to the characteristic modes propagating in the waveguide. The number of these modes can be obtained from the well known relation $M=\frac{2d}{\lambda }\sqrt{n_{c}^{2}-n_{b}^{2}}\approx 5$, where *d* stands for the thickness of the waveguiding layer.

**Figure 5.**
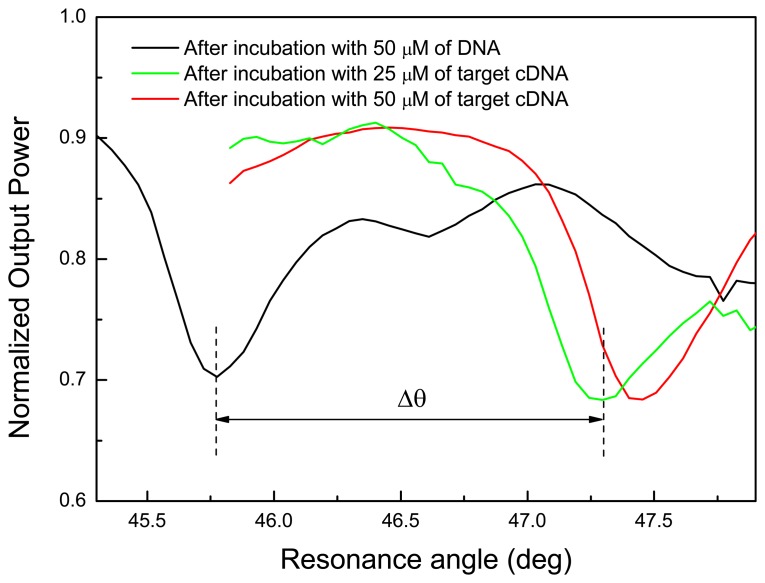
PSi RM resonances after functionalization with probe DNA and after two DNA hybridization steps: the coupling angle shifts demonstrate the molecular interaction between the probe DNA and its complementary target at different concentration.

In Figure 5, the results of angular measurements after incubation with probe DNA and after two steps of the DNA hybridization process at two different concentrations are reported: the resonance shifts confirm the effectiveness of the DNA binding.

**Figure 6.**
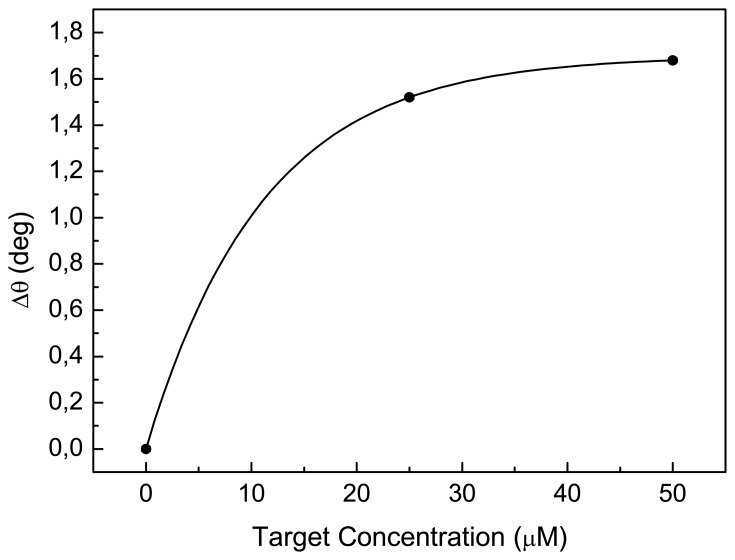
Resonance shifts of the waveguide mode coupling angle as a function of the DNA concentration.

The coupling angle shifts as a function of the concentration of the complementary DNA, as it can be seen in Figure 6. The high efficiency of hybridization is confirmed by the fact that, after exposure to 50 μM of complementary DNA, the sensor response curve reaches a plateau, corresponding to a saturation of almost all of the active sites. The experimental points can be well fitted (R^2^=0.99, χ^2^<1) by a molecular growth exponential curve: from this fit a sensitivity of 0.13±0.02 deg/μM can be estimated. Since the angular resolution of the experimental setup is 0.01 deg, we can estimate, for our measuring system, a technical limit of 77±12 nM. This value, that has been estimated from experimental data, is of the same order of magnitude of that reported in ref. [13], where a detection limit of 50 nM is predicted on the basis of numerical simulations and under the assumption of an optimal 50% probe coverage of the PSi surface. On exposure to the non complementary DNA sequence the angular resonance does not undergo any detectable shift, i.e. no shifts more than 0.01 deg can be observed. The width of the resonances (e.g. FWHM of about 0.37 deg for the first resonance) can be considerably reduced, thus increasing the sensitivity of the sensor, by reducing the waveguide losses, mainly due to the roughness of the structure. A dramatic reduction of these losses can be achieved, for example, by performing the etching process at low temperatures [14] and/or by making use of a mono-modal waveguide.

## 4. Conclusions

We have experimentally confirmed the ability of a PSi RM to detect a DNA-DNA hybridization process with very high sensitivity. The use of PSi technology in the fabrication of the waveguide, which is the heart of a RM sensor, allows the presence of a high specific-surface available for interaction with great amounts of analytes. We have demonstrated that our measuring system is characterized by a technical limit of about 80 nM of DNA, in good agreement with previously published theoretical calculations [13]. Next efforts will be aimed at the fabrication of a free-standing waveguide (no crystalline silicon substrate), which, in conjunction with a flow delivery system, could allow to reduce considerably the time of incubation of the DNA probe and targets over the functionalized surface.
